# Hypoxia Preconditioned Serum (HPS) Promotes Proliferation and Chondrogenic Phenotype of Chondrocytes In Vitro

**DOI:** 10.3390/ijms241310441

**Published:** 2023-06-21

**Authors:** Jun Jiang, Jannat Altammar, Xiaobin Cong, Lukas Ramsauer, Vincent Steinbacher, Ulf Dornseifer, Arndt F. Schilling, Hans-Günther Machens, Philipp Moog

**Affiliations:** 1 Experimental Plastic Surgery, Clinic for Plastic, Reconstructive and Hand Surgery, Klinikum Rechts der Isar, Technical University of Munich, D-81675 Munich, Germany; 2 Institute of Molecular Immunology and Experimental Oncology, Klinikum Rechts der Isar, Technical University of Munich, D-81675 Munich, Germany; 3 Department of Plastic, Reconstructive and Aesthetic Surgery, Isar Klinikum, D-80331 Munich, Germany; 4 Department of Trauma Surgery, Orthopedics and Plastic Surgery, University Medical Center Göttingen, D-37075 Göttingen, Germany

**Keywords:** autologous chondrocyte implantation, chondrocytes, dedifferentiation, peripheral blood cells, blood-derived therapy, hypoxia, hypoxia preconditioned serum, osteoarthritis, cartilage defect, regenerative medicine

## Abstract

Autologous chondrocyte implantation (ACI) for the treatment of articular cartilage defects remains challenging in terms of maintaining chondrogenic phenotype during in vitro chondrocyte expansion. Growth factor supplementation has been found supportive in improving ACI outcomes by promoting chondrocyte redifferentiation. Here, we analysed the chondrogenic growth factor concentrations in the human blood-derived secretome of Hypoxia Preconditioned Serum (HPS) and assessed the effect of HPS-10% and HPS-40% on human articular chondrocytes from osteoarthritic cartilage at different time points compared to normal fresh serum (NS-10% and NS-40%) and FCS-10% culture conditions. In HPS, the concentrations of TGF-beta1, IGF-1, bFGF, PDGF-BB and G-CSF were found to be higher than in NS. Chondrocyte proliferation was promoted with higher doses of HPS (HPS-40% vs. HPS-10%) and longer stimulation (4 vs. 2 days) compared to FCS-10%. On day 4, immunostaining of the HPS-10%-treated chondrocytes showed increased levels of collagen type II compared to the other conditions. The promotion of the chondrogenic phenotype was validated with quantitative real-time PCR for the expression of collagen type II (COL2A1), collagen type I (COL1A1), SOX9 and matrix metalloproteinase 13 (MMP13). We demonstrated the highest differentiation index (COL2A1/COL1A1) in HPS-10%-treated chondrocytes on day 4. In parallel, the expression of differentiation marker SOX9 was elevated on day 4, with HPS-10% higher than NS-10/40% and FCS-10%. The expression of the cartilage remodelling marker MMP13 was comparable across all culture conditions. These findings implicate the potential of HPS-10% to improve conventional FCS-based ACI culture protocols by promoting the proliferation and chondrogenic phenotype of chondrocytes during in vitro expansion.

## 1. Introduction

Articular cartilage facilitates movement of the joint by creating a lubricating and smooth surface [1]. The chondrocyte, a specific cell type within this avascular tissue, has a limited capacity for regeneration after injury [2]. Without immediate treatment, chondral defects can progress into larger lesions and eventually cause osteoarthritis (OA) [3]. As of 2019, global estimates suggest that up to 250 million people are affected by OA, with rising healthcare and socioeconomic costs [4].

One method of treating deep focal chondral defects of medium to large size (>2 cm^2^) is autologous chondrocyte implantation (ACI) [2,5]. ACI is a procedure that involves expanding and implanting autologous articular chondrocytes harvested from the cartilage of a non-load-bearing joint location [2,5]. The cartilage tissue created by ACI is stable and has led to satisfactory clinical results [6]. Nevertheless, yielding an adequate amount of chondrocytes is difficult due to the small biopsy volume, donor site morbidity, and the limited capacity for chondrocyte proliferation [7]. Furthermore, articular chondrocytes are predisposed to dedifferentiate towards a fibroblast-like phenotype during ACI in vitro monolayer expansion [7]. In this process, the expression of cartilage-specific collagen type II (COL2A1) decreases and fibrous collagen type I (COL1A1) expression increases [8]. Strategies to prevent dedifferentiation and promote chondrogenic redifferentiation include culture supplementation with exogenous growth factors [9,10], three-dimensional culture in biomaterials (alginate or collagen type I and/or type II) [11,12] and applying biophysical stimuli (mechanical loading and oxygen tension) [13,14]. In this context, the stimulating capabilities of growth factors have been the subject of intense research, and the following were discovered to promote chondrocyte proliferation and differentiation: TGF-beta (transforming growth factor-beta), IGF-1 (insulin-like growth factor-1), bFGF (basic fibroblast growth factor), PDGF-BB (platelet-derived growth factor BB), G-CSF (granulocyte colony-stimulating factor) and Leptin [12,15,16,17]. Among those biomolecules, TGF-beta and IGF-1 are fundamental components of signalling pathways for cartilage maintenance and regeneration, and both play crucial roles in cartilage engineering techniques [18,19]. However, recombinant growth factor supplementation comes at a high cost and the limitation of yielding sufficient chondrocytes during in vitro expansion remains [7,20]. Platelet-derived products were investigated as alternative supplementation in chondrocyte cultures due to their enriched growth factor proteins [9,21,22,23]. As such, Platelet-rich Plasma (PRP) demonstrated positive effects on chondrocytes regarding proteoglycan and collagen production, cell proliferation, and redifferentiation in vitro, as well as clinical improvement with intraarticular PRP injections for patients with degenerative joint diseases [3,9,14,24,25].

From a broader perspective, blood-derived growth factor preparations present a promising strategy of providing an accessible source of biologically complete secretomes and have attracted high interest in the field of regenerative medicine [26,27,28]. In order to advance the development of autologous growth factor preparations, we invented a newer technique using hypoxic preconditioning of peripheral blood cells (PBCs) to induce regenerative cytokines. PBCs are a natural source of physiological regeneration signals, which can be obtained by applying a particular stress-induced treatment (such as hypoxia). In this process, PBCs release biomolecules, e.g., VEGF (vascular endothelial growth factor), bFGF, PDGF-BB, into the serum compartment, which can then be filtered from blood cells (hypoxia preconditioned serum: HPS) [28,29,30,31,32,33]. As a result, HPS appears to be a promising candidate for promoting self-regeneration. In fact, we have demonstrated through a number of in vitro studies that HPS promotes various regeneration processes, including angiogenesis [28,29,30,32,34,35,36], lymphangiogenesis [37,38], fibroblast proliferation and migration [30,31,39,40], and wound healing by topically administering HPS via a hydrogel-carrier method [28,41]. Notably, there was no reduction in the angiogenic activity of HPS obtained from individuals receiving oral anticoagulation due to underlying vascular disease or those suffering from type 1 and type 2 diabetes [42]. Recently, we also effectively demonstrated the ability of HPS to promote osteoblast proliferation, migration and matrix deposition [43]. Therefore, the hypoxia-induced secretome of PBCs offers a novel method of promoting different tissue regeneration, while its effect on chondrocytes is still unknown.

Until today, ACI’s technical requirements still face the dilemma of chondrocytes’ dedifferentiation with higher cell passage versus the need for redifferentiation to preserve the ability to form cartilage tissue [7]. Based on the regenerative potential of HPS, we hypothesize that its supplementation to chondrocyte culture media can prevent or reverse dedifferentiation while maintaining appropriate cell proliferation during in vitro monolayer expansion and thus refine current ACI protocols. To verify this hypothesis, we first quantified the levels of key chondrogenic growth factors in HPS and then applied HPS of two concentrations (10% and 40%) on human articular chondrocytes from osteoarthritic cartilage. After 2 and 4 days of HPS treatment, chondrocyte proliferation, collagen type II synthesis, and the gene expression of chondrogenic markers were analysed.

## 2. Results

### 2.1. Quantitative Analysis of Chondrogenic Growth Factors in HPS

**Figure 1:**
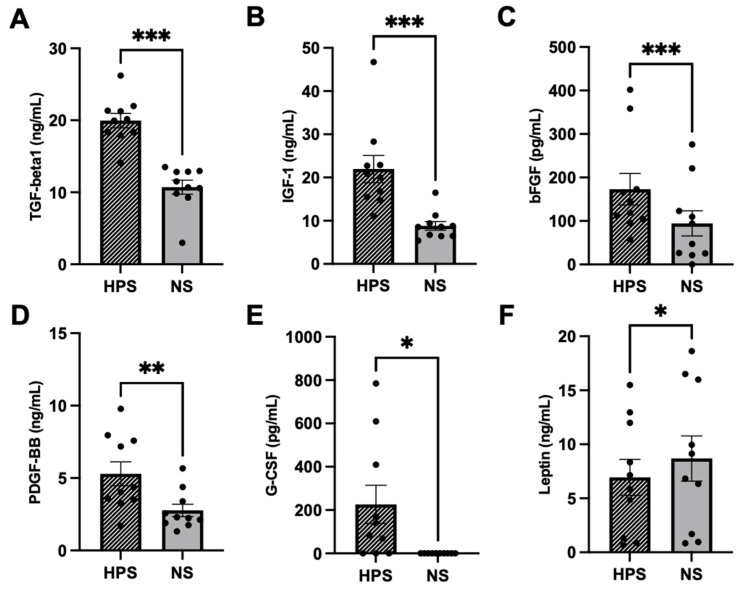
Quantitative analysis of chondrogenic growth factors in HPS and NS. (**A**) TGF-beta1, (**B**) IGF-1, (**C**) bFGF, (**D**) PDGF-BB, (**E**) G-CSF and (**F**) Leptin. The blood samples for each HPS and NS are from the same donor; blood donors: *n* = 10, paired *t*-test. Data points are means ± SEM. * *p* < 0.05, ** *p* < 0.01, *** *p* < 0.001.

Characterization of HPS regarding chondrogenic growth factors was performed by quantitative measurement of TGF-beta1, IGF-1, bFGF, PDGF-BB, G-CSF and Leptin (Figure 1). In comparison to NS, we found significantly higher levels of TGF-beta1 (*p* < 0.001), IGF-1 (*p* < 0.001), bFGF (*p* < 0.001), PDGF-BB (*p* = 0.002) and G-CSF (*p* = 0.04) in HPS. Leptin was significantly lower in HPS than in NS (*p* = 0.02).

### 2.2. The Effect of HPS on Chondrocyte Proliferation and Metabolic Activity

**Figure 2:**
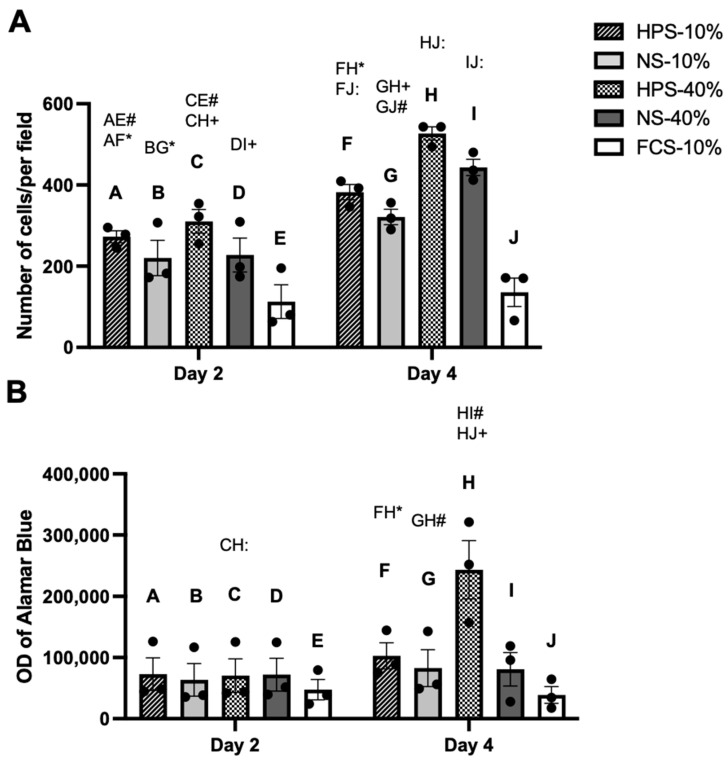
The effect of HPS on human chondrocyte proliferation and metabolic activity. Chondrocytes were stimulated by HPS-10/40% compared to NS-10/40% (pooled from ten blood donors) and FCS-10% (control) for 2 and 4 days. (**A**) Plot showing cell counts of chondrocytes per microscope field. HPS promotes cell proliferation in a time- and dose-dependent manner. (**B**) Cell metabolic activity analysed with Alamar Blue assay measured in optical density (OD). Two-way repeated-measures ANOVA with Tukey’s multiple comparisons test. Data points are means ± SEM, chondrocyte donors: *n* = 3. For each chondrocyte donor, the mean of triplicates was calculated. Capital letter pairs over plots indicate statistical comparison of corresponding data points. For all pair comparisons, * = *p* < 0.05, # = *p* < 0.01, + = *p* < 0.001, : = *p* < 0.0001.

We assessed the effects of HPS on proliferation and metabolic activity of human chondrocytes by cell counting and Alamar Blue assay. The beneficial HPS concentration was determined by analysing two HPS-dilutions (HPS-10% and HPS-40%) after 2 and 4 days of stimulation and comparing them to corresponding dilutions of NS. Here, we found a significant increase in cell numbers after 2-day stimulation by HPS-10% (2.4×) and HPS-40% (2.8×) compared to controls (*p* = 0.009 and *p* = 0.0013, respectively) (Figure 2A). Cell numbers of HPS-10/40%-treated chondrocytes were higher than their corresponding NS, but not significantly. HPS-10/40% and NS-10/40% stimulation for 4 days resulted, time-dependently, in higher chondrocyte proliferation compared to their corresponding 2-day stimulation (*p* = 0.02, *p* < 0.001, *p* = 0.04 and *p* < 0.001, respectively). On day 4, the number of chondrocytes stimulated with HPS-40% was 1.4× higher than with HPS-10% (526.7 vs. 382.3, *p* = 0.02), indicating that HPS also promotes proliferation in a dose-dependent manner. A significant increase in cell numbers after 4 days of HPS-10% (2.8×) and HPS-40% stimulation (3.9×) compared to controls was observed (382.3 vs. 135.7, *p* = 0.009 and 526.7 vs. 135.7, *p* = 0.001, respectively). Here again, cell numbers of HPS-10/40%-treated chondrocytes were higher than their corresponding NS, but not significantly.

Cell metabolic activity assessed with Alamar Blue assay showed the highest optical density (OD) values with HPS-40% stimulation on day 4 (Figure 2B). This was significantly higher than the conditions with 2 days of HPS-40% (*p* < 0.0001) and 4 days of HPS-10%, NS-10%, NS-40% stimulation and FCS-10% controls (*p* = 0.01, *p* = 0.004 and *p* < 0.001, respectively).

### 2.3. Macroscopic Cell Appearance and Immunofluorescence Analysis of Collagen Type II

**Figure 3:**
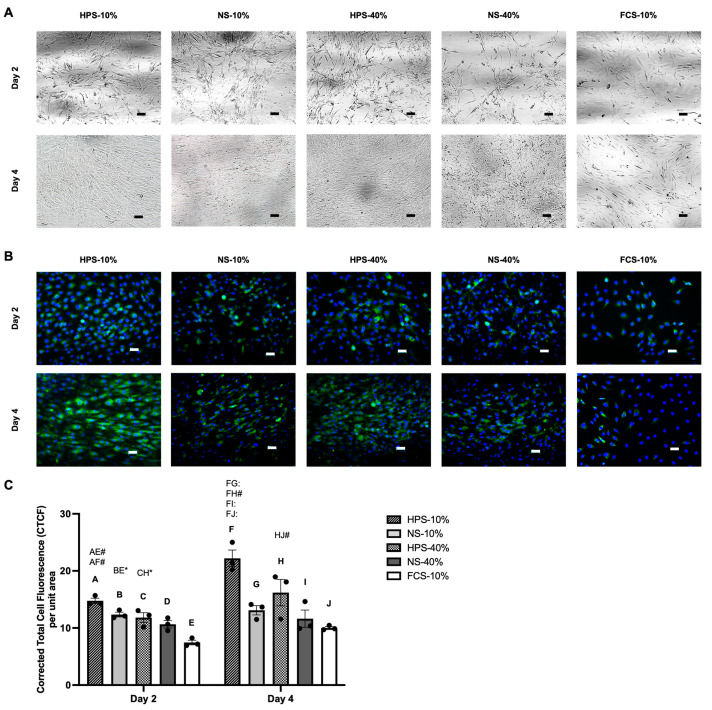
Morphology and identification of human chondrocytes treated with HPS-10/40% compared to NS-10/40% (pooled from ten blood donors) and FCS-10% (control) for 2 and 4 days. (**A**) Confocal microscopy of chondrocytes. (**B**) Immunofluorescence images of collagen type II (green) for the identification of chondrocytes counterstained with DAPI (blue). Scale bar = 50 µm. (**C**) Corrected Total Cell Fluorescence (CTCF) per unit area based on the collagen type II immunostaining depicted in (**B**). Two-way repeated-measures ANOVA with Tukey’s multiple comparisons test. Data points are means ± SEM, chondrocyte donors: *n* = 3. For each chondrocyte donor, three images per treatment condition were analysed at each time point. Capital letter pairs over plots indicate statistical comparison of corresponding data points. For all pair comparisons, * = *p* < 0.05, # = *p* < 0.01, : = *p* < 0.0001.

To assess the chondrogenic phenotype status, cultured chondrocytes were first analysed morphologically and by collagen type II immunofluorescence staining. Here, we observed an elongated/spindle-shaped cell appearance in all culture conditions on day 2 with a phase contrast microscopy (Figure 3A). Upon further proliferation until day 4, cell density and cell–cell contact started to increase. In this process, certain areas could be identified, where cells regained a more polygonal-shaped morphology of chondrocytes. This morphological switch was mostly apparent with HPS-10% stimulation. In contrast, FCS-10% chondrocyte cultures remained at a low density after 4 days of stimulation and kept a spindle-shaped morphology.

Immunostaining with collagen type II revealed a deeper insight into the chondrogenic phenotype status. On day 2, we detected various numbers of collagen type II-negative cells in different groups, as displayed by DAPI staining with no green fluorescence (Figure 3B). Here, immunofluorescence analysis showed higher signal intensities in HPS-10%- and NS-10%-treated cells than in FCS-10% controls (*p* = 0.0012 and *p* = 0.04 respectively) (Figure 3C). From 2 to 4 days of stimulation, signal intensities in HPS-10% and HPS-40% conditions increased significantly by 1.5× (*p* = 0.0013) and 1.4× (*p* = 0.044), respectively. On day 4, HPS-10%-treated chondrocytes showed the highest signal intensities, up to 2.2× higher in comparison to all other conditions. Here, numerous cells treated in NS-10/40% and FCS-10% were observed with weak/no fluorescence (Figure 3B).

### 2.4. The Effect of HPS on Chondrocyte Differentiation

**Figure 4:**
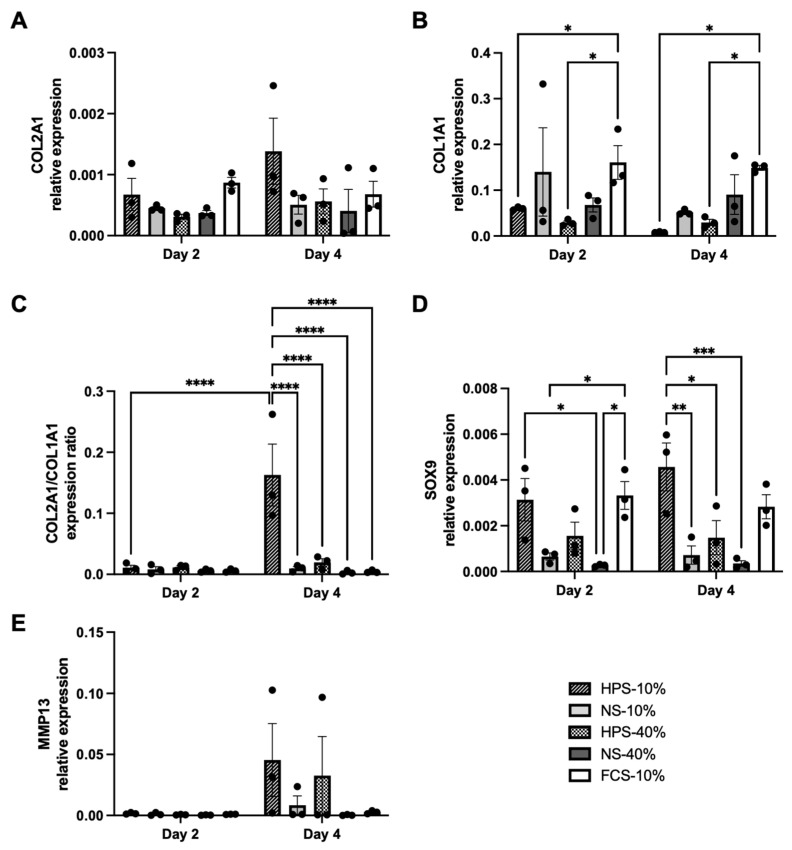
Gene expression of chondrogenic markers in human chondrocytes treated with HPS-10/40% compared to NS-10/40% (pooled from ten blood donors) and FCS-10% (control) after 2 and 4 days of stimulation. (**A**) Collagen type II (COL2A1), (**B**) collagen type I (COL1A1), (**C**) differentiation index (COL2A1/COL1A1), (**D**) SOX9 and (**E**) MMP13. Relative expression was normalized to GAPDH (dCT-method). Two-way repeated-measures ANOVA with Tukey’s multiple comparisons test. Data points are means ± SEM, chondrocyte donors: *n* = 3. For each chondrocyte donor, the mean of trials in triplicate was calculated. * *p* < 0.05, ** *p* < 0.01, *** *p* < 0.001, **** *p* < 0.0001.

In the next series of chondrogenic phenotype assessment following different culture conditions, gene expressions of differentiation (COL2A1, SOX9), dedifferentiation (COL1A1) and cartilage remodelling (MMP13) were quantified. For COL2A1, we found up to 3.4× higher expression in HPS-10% on day 4 compared to other culture conditions; however, this was not significant (Figure 4A, *p* > 0.05). For COL1A1, HPS-10% and HPS-40% already showed significantly lower expression levels on day 2 in comparison to FCS-10% (Figure 4B, *p* = 0.017 and *p* = 0.012, respectively). On day 4, COL1A1 expression in HPS-10%-treated cells decreased by 93.9% additionally, while HPS-40%-treated cells maintained their low expression levels. The chondrogenic differentiation index (COL2A1/COL1A1) was then calculated to detect chondrogenic dedifferentiation/redifferentiation processes following the different culture conditions. Here, HPS-10% displayed a significant chondrogenic redifferentiation potential, up to 53.3× higher after 4 days compared to 2 days of culture (*p* < 0.0001) and to other culture conditions at 4 days (*p* < 0.0001) (Figure 4C). Regarding the chondrogenic differentiation marker SOX9, HPS-10% increased SOX9 expression from day 2 to day 4 by 1.5×, but not significantly (*p* = 0.22). On day 4, HPS-10%-treated chondrocytes exhibited significantly higher expression of SOX9 than NS-10%-, HPS-40%- and NS-40%-treated cells (*p* = 0.002, *p* = 0.018 and *p* < 0.001, respectively). MMP13 expression was determined to assess the effects on cartilage remodelling. Here, we detected relatively higher levels of MMP13 expression on day 4 in HPS-10%/40%-treated cells compared to other conditions, but this was not significant (Figure 4E, *p* > 0.05).

## 3. Discussion

Treatments for cartilage defects have advanced dramatically in recent years, including the ACI approach [44]. Currently, the high cost of purified, genetically engineered growth factors, which are required for cellular expansion and differentiation, severely challenges the clinical use of modern ACI techniques [45]. We invented a novel and cost-effective method with HPS by harnessing a comprehensive set of growth factors released through hypoxia preconditioning of PBCs into the serum compartment. Its effect on chondrocytes was investigated in this study and compared to normal fresh serum (NS) and FCS-10% (control). Since we had previously established the safety and efficacy of HPS-10% and -40% in in vitro angiogenesis, lymphangiogenesis, osteogenesis, and in vivo skin regeneration [28,29,32,33,35,37,38,40,41,42,43], we adopted the same concentrations for a dose-dependent investigation. The results demonstrated a considerable chondrogenic response from HPS-10% treatment, including chondrocyte proliferation and differentiation, most probably by providing exogenous chondrogenic growth factors and upregulating chondrogenic gene expressions in chondrocytes.

For the chondrogenic growth factor analysis of HPS, we demonstrated approximately 2× higher levels of TGF-beta1, IGF-1, bFGF, PDGF-BB and G-CSF compared to baseline NS (Figure 1). Although Leptin is greatly influenced by the body mass index (BMI) and sex of individuals [46], we interestingly measured a significant decrease in Leptin levels in HPS in paired comparison to NS (Figure 1E). Leptin is described to promote proliferation and differentiation of chondrocytes, but in the presence of an IL-1-rich environment, such as in OA, it turns inflammatory and decreases the chondrogenic phenotype while increasing apoptosis of chondrocytes [47]. In contrast, TGF-beta1, IGF-1, PDGF-BB and G-CSF inhibit IL-1 induced catabolic inflammation and increase anabolism by upregulating the synthesis of proteoglycan, aggrecan and collagen type II [17,48,49,50]. Similarly to HPS, PRP also contains increased levels of TGF-beta1, IGF-1, bFGF, PDGF-BB and G-CSF [9,23,51,52] and exhibited chondroprotection by downregulating inflammatory markers [53,54]. In this regard, a potential anti-inflammatory effect of HPS on chondrocytes remains to be assessed via endogenous IL-1 and TNF-alpha expression analysis of chondrocytes; furthermore, a comparative evaluation of the chondrogenic growth factor composition between HPS and PRP has to be conducted in future experiments. 

It is paradoxical and simultaneously challenging to achieve sufficient cell numbers of chondrocytes which have, per se, a limited proliferation capacity [2]. Therefore, the ability to maintain the chondrogenic phenotype during chondrocyte expansion in current ACI therapy is limited [7]. One solution to this issue involves supplementing specific growth factors that aim to promote ‘redifferentiation’ from the dedifferentiated (proliferative) state by mimicking the in vivo biomolecular microenvironment [12]. This strategy presupposes a rather two-stage phenotype development in which cells initially proliferate/dedifferentiate and subsequently redifferentiate to regain their chondrogenic-specific character [12]. In terms of proliferation capacity, HPS-cultured chondrocytes proliferated more with a higher HPS concentration (HPS-40% vs. HPS-10%) and longer cultivation time (4 d vs. 2 d). Interestingly, increases in cell number and metabolic activity were not completely proportional (Figure 2A,B). Given the fact that differentiated chondrocytes are at low metabolism and less proliferative, while dedifferentiated cells are more metabolic active and proliferative [55], we postulated from this standpoint that the highly proliferative and metabolically active HPS-40%-treated cells may have dedifferentiated and lost their chondrogenic phenotype.

Cell morphology analysis can provide first insights into the chondrogenic phenotype, as chondrocytes lose their distinctive cuboid/polygonal form and undergo a morphological shift to fibroblasts during the dedifferentiation process [7]. On day 2, chondrocytes in all conditions showed spindle-shaped morphology, indicating an extensive dedifferentiation during the early proliferation stage (Figure 3A). As proliferation continued until day 4, in the HPS- and NS-treated conditions, areas of polygonal-shaped cells developed, indicating a redifferentiated chondrocyte phenotype, while FCS-treated chondrocytes remained in a spindle-shaped morphology (Figure 3A). Immunostaining of collagen type II was consistent with cell morphology and showed an increase in fluorescence with the appearance of polygonal-shaped cells (Figure 3B,C). In this regard, HPS-10% showed the strongest collagen type II signal intensity of all culture conditions on day 4 (Figure 3B,C). Conversely, NS and FCS culture conditions exhibited numerous collagen type II-negative cells, suggesting the presence of dedifferentiated chondrocytes.

The promotion of chondrogenesis at gene expression level was verified through RT-qPCR analysis of differentiation markers COL2A1 and SOX9, dedifferentiation marker COL1A1 and cartilage remodelling marker MMP13. Here, we detected an up to 3.4× higher COL2A1 expression in HPS-10%-treated chondrocytes after 4 days in comparison to other conditions; however, this was not significant (Figure 4). More importantly, COL1A1 expression diminished by 93.9% from day 2 to day 4 in HPS-10%-treated cells, resulting in a differentiation index (COL2A1/COL1A1) up to 53.3× higher than the other conditions. In parallel, SOX9 mRNA was significantly up to 13.1× higher in HPS-10%-treated cells on day 4, which validates its chondrogenic phenotype. Interestingly, it appears that the concentration of growth factors in HPS affect the chondrogenic phenotype and cell proliferation differentially: HPS-40% elicited a 67.6% lower SOX9 expression and 87.9% lower differentiation index than HPS-10%, which would indicate a dedifferentiated phenotype status, while conversely, it promoted 1.4× higher proliferation than HPS-10% on day 4. Nonetheless, when compared to the FCS-10% control, HPS-10% still promoted significant chondrocyte proliferation by up to 2.8× on both days 2 and 4. Therefore, HPS-10% seems to be beneficial in achieving sufficient chondrocyte numbers during in vitro expansion while promoting redifferentiation of the chondrogenic phenotype. We hypothesize that this is due to the dilution of anti-chondrogenic/dedifferentiating growth factors to a concentration where pro-chondrogenic factors come more to the forefront of action. This “re-balancing” of growth factors was previously demonstrated by lower concentrations of HPS-10%, which exhibited a greater regenerative effect than higher concentrations of HPS-40% in lymphendothelial cell sprouting, vascular cell sprouting and osteoblast migration [28,35,37,43]. From another perspective, it would be intriguing to elucidate whether HPS-40%-dedifferentiated chondrocytes can be redifferentiated with an interchange to HPS-10% culture, since chondrocytes undergo reversible dedifferentiation when exposed to greater doses of bFGF (as in HPS-40%) [12]. It has to be noted that MMP13 was increased in HPS-10/40%-treated cells on day 4, but not significantly. MMP13 is a potentially destructive proteinase causing cartilage breakdown and its overexpression is associated with OA, but it was also shown to play an essential role in physiologic cartilage remodelling [56]. Additionally, MMP13 is required for chondrocyte differentiation due to its involvement in SOX9 expression [57]. This is consistent with our findings which indicated that upregulation of SOX9 in HPS-10%-treated cells correlated with an increased trend towards MMP13 expression. Altogether, further investigations on the chondrogenic effect of more serial HPS dilutions and assessing additional de-/differentiation markers, e.g., aggrecan and collagen type X, would be of high interest in order to evaluate more precisely the impact of HPS on chondrocyte differentiation and cartilage matrix production. 

In comparison to studies of platelet-derived secretomes, PRP stimulated 2D monolayer proliferation as well, while also inducing redifferentiation via decreased COL1A1 and increased COL2A1 expression [23,24], particularly in hypoxic 1% O_2_ culture conditions, which resulted in 10× higher COL2A1 expression and COL2A1/COL1A1 differentiation index in comparison to normoxia (20% O_2_) [9]. The benefit of hypoxia in chondrocyte culture is known to resemble the native anaerobic microenvironment in joints during cartilage remodelling [58]. In such an environment, the hypoxia-inducible factor (HIF) is engaged to increase chondrocyte metabolism, upregulate chondrogenic marker expression, and promote matrix synthesis [58,59]. Whether HPS causes equivalent hypoxia signalling via the HIF pathway in chondrocytes is still unknown. Similarly, the hypoxia preconditioned media derived from mesenchymal stem cells (MSCs) also demonstrated pro-chondrogenic effects by provision of soluble growth factors and more specifically by extracellular vesicle-mediated microRNA delivery [60,61]. This hypoxia-stimulated paracrine mechanism may also be involved in hypoxic preconditioning of PBCs and subsequently activate the related chondrogenic signalling pathways in chondrocytes, however, further research is required.

As the ACI technique evolves, adaptions of the culture conditions (growth factors, PRP), cell sources (MSCs, induced pluripotent stem cells, adipose-derived stem cells) and application of scaffolds (natural, synthetic) have been developed [7]. The latter has resulted in the development of the next-generation Matrix-assisted ACI (MACI), which involves in vitro chondrocyte expansion in a native three-dimensional (3D) configuration. This approach has been considered as more efficient than the simple 3D pellet culture which is limited in clinical translation due to the small pellet size and difficulties in transplantation [54]. In fact, the 3D matrix structure can also be created with HPS by adding thrombin/calcium/fibrinogen to form a fibrin matrix, in which chondrocytes can be seeded for in vitro expansion. Our previous studies have already demonstrated the benefits of HPS fibrin in terms of vascular endothelial cell angiogenesis [28,29,32]. It would be promising to verify a pro-chondrogenic ability of HPS-fibrin on chondrocytes in further studies, particularly in comparison to Platelet-rich Fibrin (PRF), which has been shown to be effective for chondrocyte implantation both in vitro and in vivo [62].

We acknowledge that the small size of chondrocyte donors (*n* = 3) is a limitation of this study. However, relevant findings were obtained and the inter-donor variation was reduced with the use of ten pooled blood donors.

## 4. Materials and Methods

### 4.1. Ethical Approval

This study was conducted as per the Declaration of Helsinki and the approval of the ethics committee of the Technical University Munich, Germany (File Nr.: 49/21S; date of approval: 27 January 2021). Informed consent was obtained from all blood donors involved.

### 4.2. Production of Hypoxia Preconditioned Serum (HPS)

The production of HPS followed the protocol previously described by our group [28]. Ten healthy human donors, comprised of five females and five males, aged between 23 and 39, were involved. Exclusion criteria were the following: smoking, pregnancy, systemic inflammatory diseases, and any oral medication taken within 6 weeks prior to blood donation. Briefly, we collected 20 mL of the peripheral venous blood into a 30 mL syringe (Omnifix^®^, B Braun AG, Melsungen, Germany), and added 5 mL of air through a 0.2 µm filter (Sterifix^®^, B Braun AG, Melsungen, Germany). We sealed the syringe and placed it for 4 days at 37 °C in a widely available 5% CO_2_ incubator (the choice of CO_2_ amount has no relevance). In this cultivation period, pericellular hypoxia (∼1% O_2_) is created by oxygen consumption of the PBCs with a blood volume per unit area (BVUA) > 1 mL/cm^2^, as previously demonstrated [32,34]. A top serum layer (HPS) was formed after 4 days by PBC sedimentation and blood coagulation and was filtered (Sterifix^®^, B Braun AG, Melsungen, Germany) into another syringe. The resulting HPS was stored in individual or pooled aliquots at −80 °C until experimental testing, with a maximum storage time of 3 months.

### 4.3. Production of Fresh Normal Serum (NS)

The donors of NS were identical to those of HPS, as described in Section 4.2. For NS production, we collected 20 mL of peripheral venous blood into 30 mL syringes (Omnifix^®^, B Braun AG, Melsungen, Germany). The syringes stood upright for 4 h at room temperature to allow simple sedimentation. The top serum layer (NS) was filtered (Sterifix^®^, B Braun AG, Melsungen, Germany) into another syringe and was freshly frozen at −80 °C in individual or pooled aliquots until experimental testing, with a maximum storage time of 3 months. 

### 4.4. Cell Culture

The Primary Human Chondrocytes (HCH) isolated from osteoarthritic cartilage of the hip joints from three male donors (58.33 ± 6.13 years) were purchased from PromoCell (C-12750, PromoCell GmbH, Heidelberg, Germany). Cells were cultured in Chondrocyte Growth Medium (C-27111, PromoCell GmbH, Heidelberg, Germany) containing Growth Medium SupplementMix (C-39635, PromoCell GmbH, Heidelberg, Germany) and 1% Antibiotic/Antimycotic Solution (ab/am) (100× solution with 10,000 units/mL penicillin and 10 mg/mL streptomycin, BioReagent, Sigma-Aldrich Chemie GmbH, Taufkirchen, Germany) at 37 °C and 5% CO_2_. All the following experiments were conducted at the 4–6th cellular passages. For the HPS/NS culture groups, the frozen HPS/NS were thawed and diluted to a final concentration of 10% and 40% using DMEM (PAN-Biotech GmbH, Aidenbach, Germany) and 1% ab/am. For the control medium, 10% FCS (Biochrom GmbH, Berlin, Germany) was prepared in DMEM with 1% ab/am. Culture media (HPS, NS and the control media) were not changed over the course of each experiment.

### 4.5. Quantification of the Chondrogenic Cytokines

Respective ELISA (enzyme-linked immunosorbent assay) kits were used to quantify the levels of chondrogenic cytokines (TGF-beta1, IGF-1, bFGF, PDGF-BB, Leptin and G-CSF) in HPS and NS from individual donors (*n* = 10). The specific ELISAs (DY240 for TGF-beta1, DY291 for IGF-1, DY233 for bFGF, DY220 for PDGF-BB, DY398 for Leptin, DY214 for G-CSF, DuoSet, Bio-Techne Ltd., Minneapolis, MN, USA) were performed following the manufacturer’s instructions. OD was measured at a 450 nm wavelength using the Mithras LB 940 Multimode Microplate Reader (Berthold Technologies GmbH & Co. KG, Bad Wildbad, Germany).

### 4.6. Alamar Blue Metabolic Assay

The metabolic activity of chondrocytes was assessed using the Alamar Blue assay. Chondrocytes were initially seeded on 96-well plates at a density of 15,000/cm^2^ in 200 µL of the Chondrocyte Growth Medium per well and allowed to adhere overnight. On the following day, media were changed to 200 µL of HPS/NS-10/40% (from ten pooled blood donors) and FCS-10% and the chondrocytes were incubated for 2 and 4 days on separate plates. At each timepoint, cell culture media were removed and replaced with 200 µL of Alamar Blue solution (Resazurin, Sigma-Aldrich, St. Louis, MO, USA) diluted with Chondrocyte Growth Medium at a ratio of 1:10. The plates were then incubated in the incubator at 37 ℃ for 2 h. After incubation, 100 µL per well of the supernatant was transferred to another 96-well plate. The optical intensity was measured with an excitation wavelength of 560 nm, emission wavelength of 590 nm, and reference wavelength of 629 nm using the Mithras LB 940 Multimode Microplate Reader (Berthold Technologies GmbH & Co. KG, Bad Wildbad, Germany). For each chondrocyte donor (*n* = 3), the mean of the trials conducted in triplicate was calculated.

### 4.7. Immunofluorescence Staining

The cultured chondrocytes were analysed by immunochemistry to detect the production of type II collagen. Titration of the antibodies were performed to determine their optimal concentrations. On day 2 and day 4, respectively, the samples were fixed with 3.7% formaldehyde (AppliChem, Darmstadt, Germany) at room temperature for 20 min, followed by washing with PBS (Pan Biotech, Aidenbach, Germany). Samples were preserved at 4 °C until further processing, in which the fixed cells were washed three times with PBS and blocked with a solution of 1% BSA (Sigma-Aldrich, Stockholm, Sweden) and 10% normal goat serum (Abcam, Cambridge, UK) in PBS for one hour at room temperature. The rabbit anti-human Col-II antibodies (ab34712, Abcam, Cambridge, UK) were then added at 1:100 dilution with 1% BSA in PBS and were incubated overnight at 4°C. The negative controls were incubated with dilution buffer without primary antibodies. The next day, the Alexa Fluor^®^ 488-conjugated goat anti-rabbit IgG H&L antibodies (Abcam, Cambridge, UK) were added at 1:500 dilution and were incubated at room temperature for 1 h. The nuclei were counterstained using a 1:20,000 dilution of DAPI solution (D3571, Molecular probes, Eugene, OR, USA) for a duration of 1 h. Finally, the samples were visualized and imaged using an inverted fluorescence microscope (Zeiss Axio Observer Z1, Germany).

### 4.8. Quantification of Cell Number and Fluorescence Intensity

The quantification of cell number and fluorescence intensity was carried out using the ImageJ software (version 1.52, National Institutes of Mental Health, Bethesda, MD, USA), described previously in published methods [63,64]. In brief, DAPI-stained nuclei were sharpened by bandpass filtering and visually identified on each image using threshold settings. These threshold-processed nuclei were automatically recognized and counted using the built-in “Analyze Particles” feature to obtain the cell number. For fluorescence intensity, ten cells were randomly selected, and their total fluorescence was quantified using the built-in “Measure” tool. The final corrected cell fluorescence (CCF) was measured as corrected total cell fluorescence (CTCF) per unit area (CTCF/pixel^2^):

CCF = (Integrated Density − (Area of cell × Mean fluorescence of background readings))/Area of cell.(1)

For each donor, three images per treatment condition were analysed at each time point.

### 4.9. Analysis of Gene Expression

Gene expressions of chondrogenic (COL2A1, SOX9), fibrous (COL1A1) and cartilage remodelling (MMP13) markers were evaluated by real-time quantitative reverse transcription-polymerase chain reaction (RT-qPCR) for phenotype analysis. Glyceraldehyde 3-phosphate dehydrogenase (GAPDH) was selected as the reference housekeeping gene for all gene expression analyses. On day 2 and day 4, the samples were lysed with Buffer RLT lysis buffer (QIAGEN GmbH, Hilden, Germany) and stored at −80 °C until further processing. The RNeasy Mini Kit (QIAGEN GmbH, Hilden, Germany) was used to extract total RNA following the manufacturer’s instructions. The purity and concentration of RNA were determined using a NanoDrop spectrophotometer (Implen GmbH, Munich, Germany). The reverse transcription of RNA samples was performed using the SensiFast cDNA Synthesis Kit (Meridian Bioscience, Cincinnati, OH, USA) according to the manufacturer’s instructions with 500 ng of total RNA. The generated cDNA was used in triplicates for RT-PCR reactions, with primers that span exon–exon boundaries used to ensure cDNA-specific amplification. The qPCR was performed using the No ROX SYBR MasterMix blue dTTP (Eurogentec, Lüttich, Belgium). Briefly, the sample was mixed with the PCR mastermix and gene-specific primer mix in a 384-well plate (Fisher-Scientific, Waltham, MA, USA) with a final primer concentration of 100 nM. The plate was sealed and mixed by vortexing, followed by centrifugation at 2000× *g* for 5 min at 4 °C. The plate was then placed in a Roche Light Cycler 480 II (Roche, Basel, Switzerland) and was run with the following 3-step program: Carryover prevention at 50 °C for 2 min, denaturation and activation at 95 °C for 5 min, followed by 40 cycles of denaturation at 95 °C for 10 s, annealing at 62 °C for 20 s and extension at 72 °C for 30 s. Correct qPCR product size was assessed by melting curve analysis and subsequent agarose gel electrophoresis. All data were normalized to GAPDH and analysed using the dCT method. All primers are listed in Table 1.

### 4.10. Statistical Analysis

Data sets were analysed by paired *t*-test if two comparison groups are available. If more than two groups are present, a repeated-measures one-way analysis of variance (RM-ANOVA), with subsequent comparisons using Tukey’s post hoc analysis was performed. All values are expressed as means ± standard error of the mean (SEM). A value of *p* < 0.05 was considered statistically significant (* *p* < 0.05, ** *p* < 0.01, *** *p* < 0.001, and **** *p* < 0.0001).

## 5. Conclusions

Chondrocyte culture supplementation with HPS-10% has been established to be beneficial in both proliferation and chondrogenic differentiation during in vitro expansion. This observed effect is probably due to its balanced composition of chondrogenic growth factors, which contributed to a greater collagen type II immunostaining, higher differentiation ratio (COL2A1/COL1A1) and upregulation of chondrogenic SOX9 expression. By utilizing this autologous and cost-effective approach, current ACI protocols with bovine and recombinant growth factors could therefore be updated. Nevertheless, further investigation is needed to determine its in vivo efficacy in cartilage repair. 

## 6. Patents

Device-based methods for localized delivery of cell-free carriers with stress-induced cellular factors. (AU2013214187 (B2); 9 February 2017): Schilling Arndt, Hadjipanayi Ektoras, Machens Hans-Günther.

## Figures and Tables

**Table 1:** Primer sequences.

Target Gene	Primer Forward	Primer Reverse
GAPDH	CTCTGCTCCTCCTGTTCGAC	ACGACCAAATCCGTTGACTC
COL2A1	GTGTCAGGGCCAGGATGT	TCCCAGTGTCACAGACACAGAT
COL1A1	GGGATTCCCTGGACCTAAAG	GGAACACCTCGCTCTCCAG
SOX9	TACCCGCACTTGCACAAC	TCTCGCTCTCGTTCAGAAGTC
MMP13	TTTCCTCCTGGGCCAAAT	GCAACAAGAAACAAGTTGTAGCC

## Data Availability

The data presented in this study are available on request from the corresponding authors.

## Abbreviations

ACI	Autologous chondrocyte implantation
bFGF	Basic fibroblast growth factor
COL1A1	Collagen type 1 alpha 1
COL2A1	Collagen type 2 alpha 1
G-CSF	Granulocyte colony-stimulating factor
GAPDH	Glyceraldehyde 3-phosphate dehydrogenase
HPS	Hypoxia Preconditioned Serum
IGF-1	Insulin-like growth factor-1
IL-1	Interleukin-1
MMP-13	Matrix metalloproteinase-13
PBC	Peripheral blood cells
PBS	Phosphate-buffered saline
PDGF-BB	Platelet-derived growth factor-beta polypeptide
PRP	Platelet-rich plasma
SOX9	Sex-determining region Y-box 9
RT-qPCR	Real-time quantitative reverse transcription-polymerase chain reaction
TGF-beta1	Transforming growth factor-beta 1
TNF-alpha	Tumour necrosis factor-alpha

## References

[1] Sophia Fox A.J., Bedi A., Rodeo S.A. (2009). The basic science of articular cartilage: Structure, composition, and function. Sports Health.

[2] Chiang H., Jiang C.-C. (2009). Repair of Articular Cartilage Defects: Review and Perspectives. J. Formos. Med. Assoc..

[3] Bedi A., Feeley B.T., Williams R.J. (2010). Management of articular cartilage defects of the knee. J. Bone Joint Surg. Am..

[4] Hunter D.J., Bierma-Zeinstra S. (2019). Osteoarthritis. Lancet.

[5] Liu Y., Shah K.M., Luo J. (2021). Strategies for Articular Cartilage Repair and Regeneration. Front. Bioeng. Biotechnol..

[6] Ogura T., Bryant T., Minas T. (2017). Long-term Outcomes of Autologous Chondrocyte Implantation in Adolescent Patients. Am. J. Sports Med..

[7] Davies R.L., Kuiper N.J. (2019). Regenerative Medicine: A Review of the Evolution of Autologous Chondrocyte Implantation (ACI) Therapy. Bioengineering.

[8] Schnabel M., Marlovits S., Eckhoff G., Fichtel I., Gotzen L., Vécsei V., Schlegel J. (2002). Dedifferentiation-associated changes in morphology and gene expression in primary human articular chondrocytes in cell culture. Osteoarthr. Cartil..

[9] Jeyakumar V., Niculescu-Morzsa E., Bauer C., Lacza Z., Nehrer S. (2017). Platelet-Rich Plasma Supports Proliferation and Redifferentiation of Chondrocytes during In Vitro Expansion. Front. Bioeng. Biotechnol..

[10] Pei M., Seidel J., Vunjak-Novakovic G., Freed L.E. (2002). Growth factors for sequential cellular de- and re-differentiation in tissue engineering. Biochem. Biophys. Res. Commun..

[11] Aurich M., Hofmann G.O., Best N., Rolauffs B. (2018). Induced Redifferentiation of Human Chondrocytes from Articular Cartilage Lesion in Alginate Bead Culture After Monolayer Dedifferentiation: An Alternative Cell Source for Cell-Based Therapies?. Tissue Eng. A.

[12] Hu X., Zhang W., Li X., Zhong D., Li Y., Li J., Jin R. (2021). Strategies to Modulate the Redifferentiation of Chondrocytes. Front. Bioeng. Biotechnol..

[13] Duval E., Leclercq S., Elissalde J.M., Demoor M., Galéra P., Boumédiene K. (2009). Hypoxia-inducible factor 1alpha inhibits the fibroblast-like markers type I and type III collagen during hypoxia-induced chondrocyte redifferentiation: Hypoxia not only induces type II collagen and aggrecan, but it also inhibits type I and type III collagen in the hypoxia-inducible factor 1alpha-dependent redifferentiation of chondrocytes. Arthritis Rheum..

[14] Pötter N., Westbrock F., Grad S., Alini M., Stoddart M.J., Schmal H., Kubosch D., Salzmann G., Kubosch E.J. (2021). Evaluation of the influence of platelet-rich plasma (PRP), platelet lysate (PL) and mechanical loading on chondrogenesis in vitro. Sci. Rep..

[15] Brandl A., Angele P., Roll C., Prantl L., Kujat R., Kinner B. (2010). Influence of the growth factors PDGF-BB, TGF-beta1 and bFGF on the replicative aging of human articular chondrocytes during in vitro expansion. J. Orthop. Res..

[16] Liang J., Feng J., Wu W.K., Xiao J., Wu Z., Han D., Zhu Y., Qiu G. (2011). Leptin-mediated cytoskeletal remodeling in chondrocytes occurs via the RhoA/ROCK pathway. J. Orthop. Res..

[17] Marmotti A., Bonasia D.E., Bruzzone M., Rossi R., Castoldi F., Collo G., Realmuto C., Tarella C., Peretti G.M. (2013). Human cartilage fragments in a composite scaffold for single-stage cartilage repair: An in vitro study of the chondrocyte migration and the influence of TGF-β1 and G-CSF. Knee Surg. Sports Traumatol. Arthrosc..

[18] Jakobsen R.B., Østrup E., Zhang X., Mikkelsen T.S., Brinchmann J.E. (2014). Analysis of the effects of five factors relevant to in vitro chondrogenesis of human mesenchymal stem cells using factorial design and high throughput mRNA-profiling. PLoS ONE.

[19] Mullen L.M., Best S.M., Ghose S., Wardale J., Rushton N., Cameron R.E. (2015). Bioactive IGF-1 release from collagen-GAG scaffold to enhance cartilage repair in vitro. J. Mater. Sci. Mater. Med..

[20] Vonk L.A., Roël G., Hernigou J., Kaps C., Hernigou P. (2021). Role of Matrix-Associated Autologous Chondrocyte Implantation with Spheroids in the Treatment of Large Chondral Defects in the Knee: A Systematic Review. Int. J. Mol. Sci..

[21] Drengk A., Zapf A., Stürmer E.K., Stürmer K.M., Frosch K.H. (2009). Influence of platelet-rich plasma on chondrogenic differentiation and proliferation of chondrocytes and mesenchymal stem cells. Cells Tissues Organs.

[22] Okuda K., Kawase T., Momose M., Murata M., Saito Y., Suzuki H., Wolff L.F., Yoshie H. (2003). Platelet-rich plasma contains high levels of platelet-derived growth factor and transforming growth factor-beta and modulates the proliferation of periodontally related cells in vitro. J. Periodontol..

[23] Spreafico A., Chellini F., Frediani B., Bernardini G., Niccolini S., Serchi T., Collodel G., Paffetti A., Fossombroni V., Galeazzi M. (2009). Biochemical investigation of the effects of human platelet releasates on human articular chondrocytes. J. Cell. Biochem..

[24] Akeda K., An H.S., Okuma M., Attawia M., Miyamoto K., Thonar E.J., Lenz M.E., Sah R.L., Masuda K. (2006). Platelet-rich plasma stimulates porcine articular chondrocyte proliferation and matrix biosynthesis. Osteoarthr. Cartil..

[25] Gobbi A., Lad D., Karnatzikos G. (2015). The effects of repeated intra-articular PRP injections on clinical outcomes of early osteoarthritis of the knee. Knee Surg. Sports Traumatol. Arthrosc..

[26] Alsousou J., Ali A., Willett K., Harrison P. (2013). The role of platelet-rich plasma in tissue regeneration. Platelets.

[27] Lang S., Loibl M., Herrmann M. (2018). Platelet-Rich Plasma in Tissue Engineering: Hype and Hope. Eur. Surg. Res..

[28] Hadjipanayi E., Moog P., Bekeran S., Kirchhoff K., Berezhnoi A., Aguirre J., Bauer A.T., Kukrek H., Schmauss D., Hopfner U. (2019). In Vitro Characterization of Hypoxia Preconditioned Serum (HPS)-Fibrin Hydrogels: Basis for an Injectable Biomimetic Tissue Regeneration Therapy. J. Funct. Biomater..

[29] Hadjipanayi E., Bauer A.T., Moog P., Salgin B., Kuekrek H., Fersch B., Hopfner U., Meissner T., Schluter A., Ninkovic M. (2013). Cell-free carrier system for localized delivery of peripheral blood cell-derived engineered factor signaling: Towards development of a one-step device for autologous angiogenic therapy. J. Control. Release.

[30] Hadjipanayi E., Cheema U., Hopfner U., Bauer A., Machens H.G., Schilling A.F. (2012). Injectable system for spatio-temporally controlled delivery of hypoxia-induced angiogenic signalling. J. Control. Release.

[31] Hadjipanayi E., Cheema U., Mudera V., Deng D., Liu W., Brown R.A. (2011). First implantable device for hypoxia-mediated angiogenic induction. J. Control. Release.

[32] Hadjipanayi E., Kuhn P.H., Moog P., Bauer A.T., Kuekrek H., Mirzoyan L., Hummel A., Kirchhoff K., Salgin B., Isenburg S. (2015). The Fibrin Matrix Regulates Angiogenic Responses within the Hemostatic Microenvironment through Biochemical Control. PLoS ONE.

[33] Hadjipanayi E., Bekeran S., Moog P. (2018). Extracorporeal Wound Simulation as a Foundation for Tissue Repair and Regeneration Therapies. Int. J. Transplant. Plast. Surg..

[34] Hadjipanayi E., Schilling A.F. (2014). Regeneration through autologous hypoxia preconditioned plasma. Organogenesis.

[35] Moog P., Kirchhoff K., Bekeran S., Bauer A.T., von Isenburg S., Dornseifer U., Machens H.G., Schilling A.F., Hadjipanayi E. (2020). Comparative Evaluation of the Angiogenic Potential of Hypoxia Preconditioned Blood-Derived Secretomes and Platelet-Rich Plasma: An In Vitro Analysis. Biomedicines.

[36] Moog P., Hughes J., Jiang J., Röper L., Dornseifer U., Schilling A.F., Machens H.-G., Hadjipanayi E. (2023). Comparison of the Effect of Different Conditioning Media on the Angiogenic Potential of Hypoxia Preconditioned Blood-Derived Secretomes: Towards Engineering Next-Generation Autologous Growth Factor Cocktails. Int. J. Mol. Sci..

[37] Jiang J., Cong X., Alageel S., Dornseifer U., Schilling A.F., Hadjipanayi E., Machens H.-G., Moog P. (2023). In Vitro Comparison of Lymphangiogenic Potential of Hypoxia Preconditioned Serum (HPS) and Platelet-Rich Plasma (PRP). Int. J. Mol. Sci..

[38] Moog P., Schams R., Schneidinger A., Schilling A.F., Machens H.G., Hadjipanayi E., Dornseifer U. (2020). Effect of Hypoxia Preconditioned Secretomes on Lymphangiogenic and Angiogenic Sprouting: An in Vitro Analysis. Biomedicines.

[39] Hadjipanayi E., Brown R.A., Mudera V., Deng D., Liu W., Cheema U. (2010). Controlling physiological angiogenesis by hypoxia-induced signaling. J. Control. Release.

[40] Hadjipanayi E., Schilling A.F. (2013). Hypoxia-based strategies for angiogenic induction: The dawn of a new era for ischemia therapy and tissue regeneration. Organogenesis.

[41] Jiang J., Kraneburg U., Dornseifer U., Schilling A.F., Hadjipanayi E., Machens H.G., Moog P. (2022). Hypoxia Preconditioned Serum (HPS)-Hydrogel Can Accelerate Dermal Wound Healing in Mice-An In Vivo Pilot Study. Biomedicines.

[42] Moog P., Jensch M., Hughes J., Salgin B., Dornseifer U., Machens H.G., Schilling A.F., Hadjipanayi E. (2020). Use of Oral Anticoagulation and Diabetes Do Not Inhibit the Angiogenic Potential of Hypoxia Preconditioned Blood-Derived Secretomes. Biomedicines.

[43] Jiang J., Röper L., Alageel S., Dornseifer U., Schilling A.F., Hadjipanayi E., Machens H.-G., Moog P. (2022). Hypoxia Preconditioned Serum (HPS) Promotes Osteoblast Proliferation, Migration and Matrix Deposition. Biomedicines.

[44] Colombini A., Libonati F., Lopa S., Peretti G.M., Moretti M., de Girolamo L. (2022). Autologous chondrocyte implantation provides good long-term clinical results in the treatment of knee osteoarthritis: A systematic review. Knee Surg. Sports Traumatol. Arthrosc..

[45] Xiang Y., Bunpetch V., Zhou W., Ouyang H. (2019). Optimization strategies for ACI: A step-chronicle review. J. Orthop. Transl..

[46] Chow V.T., Phoon M.C. (2003). Measurement of serum leptin concentrations in university undergraduates by competitive ELISA reveals correlations with body mass index and sex. Adv. Physiol. Educ..

[47] Otero M., Lago R., Lago F., Reino J.J.G., Gualillo O. (2005). Signalling pathway involved in nitric oxide synthase type II activation in chondrocytes: Synergistic effect of leptin with interleukin-1. Arthritis Res. Ther..

[48] Quintero M., Colantuoni G., Khatib A.M., Panasyuk A., Lomri A., Mitrovic D.R. (2001). Granulocyte-macrophage colony stimulating factor activates proteoglycan, type II collagen, and cAMP production by rat articular chondrocytes through specific binding sites. J. Rheumatol..

[49] Montaseri A., Busch F., Mobasheri A., Buhrmann C., Aldinger C., Rad J.S., Shakibaei M. (2011). IGF-1 and PDGF-bb suppress IL-1β-induced cartilage degradation through down-regulation of NF-κB signaling: Involvement of Src/PI-3K/AKT pathway. PLoS ONE.

[50] Vuolteenaho K., Moilanen T., Jalonen U., Lahti A., Nieminen R., van Beuningen H.M., van der Kraan P.M., Moilanen E. (2005). TGFbeta inhibits IL-1-induced iNOS expression and NO production in immortalized chondrocytes. Inflamm. Res..

[51] El-Sharkawy H., Kantarci A., Deady J., Hasturk H., Liu H., Alshahat M., Van Dyke T.E. (2007). Platelet-rich plasma: Growth factors and pro- and anti-inflammatory properties. J. Periodontol..

[52] Qian Y., Han Q., Chen W., Song J., Zhao X., Ouyang Y., Yuan W., Fan C. (2017). Platelet-Rich Plasma Derived Growth Factors Contribute to Stem Cell Differentiation in Musculoskeletal Regeneration. Front. Chem..

[53] Moussa M., Lajeunesse D., Hilal G., El Atat O., Haykal G., Serhal R., Chalhoub A., Khalil C., Alaaeddine N. (2017). Platelet rich plasma (PRP) induces chondroprotection via increasing autophagy, anti-inflammatory markers, and decreasing apoptosis in human osteoarthritic cartilage. Exp. Cell Res..

[54] Jeyakumar V., Niculescu-Morzsa E., Bauer C., Lacza Z., Nehrer S. (2019). Redifferentiation of Articular Chondrocytes by Hyperacute Serum and Platelet Rich Plasma in Collagen Type I Hydrogels. Int. J. Mol. Sci..

[55] Zheng L., Zhang Z., Sheng P., Mobasheri A. (2021). The role of metabolism in chondrocyte dysfunction and the progression of osteoarthritis. Ageing Res. Rev..

[56] Yamamoto K., Okano H., Miyagawa W., Visse R., Shitomi Y., Santamaria S., Dudhia J., Troeberg L., Strickland D.K., Hirohata S. (2016). MMP-13 is constitutively produced in human chondrocytes and co-endocytosed with ADAMTS-5 and TIMP-3 by the endocytic receptor LRP1. Matrix Biol..

[57] Borzí R.M., Olivotto E., Pagani S., Vitellozzi R., Neri S., Battistelli M., Falcieri E., Facchini A., Flamigni F., Penzo M. (2010). Matrix metalloproteinase 13 loss associated with impaired extracellular matrix remodeling disrupts chondrocyte differentiation by concerted effects on multiple regulatory factors. Arthritis Rheum..

[58] Murphy C.L., Thoms B.L., Vaghjiani R.J., Lafont J.E. (2009). Hypoxia. HIF-mediated articular chondrocyte function: Prospects for cartilage repair. Arthritis Res. Ther..

[59] Li H., Li X., Jing X., Li M., Ren Y., Chen J., Yang C., Wu H., Guo F. (2018). Hypoxia promotes maintenance of the chondrogenic phenotype in rat growth plate chondrocytes through the HIF-1α/YAP signaling pathway. Int. J. Mol. Med..

[60] Yang Y., Wu Y., Yang D., Neo S.H., Kadir N.D., Goh D., Tan J.X., Denslin V., Lee E.H., Yang Z. (2023). Secretive derived from hypoxia preconditioned mesenchymal stem cells promote cartilage regeneration and mitigate joint inflammation via extracellular vesicles. Bioact. Mater..

[61] Zhang B., Tian X., Qu Z., Hao J., Zhang W. (2022). Hypoxia-Preconditioned Extracellular Vesicles from Mesenchymal Stem Cells Improve Cartilage Repair in Osteoarthritis. Membranes.

[62] Barbon S., Stocco E., Macchi V., Contran M., Grandi F., Borean A., Parnigotto P.P., Porzionato A., De Caro R. (2019). Platelet-Rich Fibrin Scaffolds for Cartilage and Tendon Regenerative Medicine: From Bench to Bedside. Int. J. Mol. Sci..

[63] Nichele L., Persichetti V., Lucidi M., Cincotti G. (2020). Quantitative evaluation of ImageJ thresholding algorithms for microbial cell counting. OSA Contin..

[64] Bora P., Gahurova L., Mašek T., Hauserova A., Potěšil D., Jansova D., Susor A., Zdráhal Z., Ajduk A., Pospíšek M. (2021). p38-MAPK-mediated translation regulation during early blastocyst development is required for primitive endoderm differentiation in mice. Commun. Biol..

